# Long-Term Exposure to Urban Air Pollution and Mortality in a Cohort of More than a Million Adults in Rome

**DOI:** 10.1289/ehp.1205862

**Published:** 2013-01-08

**Authors:** Giulia Cesaroni, Chiara Badaloni, Claudio Gariazzo, Massimo Stafoggia, Roberto Sozzi, Marina Davoli, Francesco Forastiere

**Affiliations:** 1 Department of Epidemiology, Lazio Regional Health Service, Rome, Italy; 2 Italian Workers’ Compensation Authority (INAIL), Rome, Italy; 3 Regional Environmental Protection Agency, Rome, Italy

**Keywords:** air pollution, cardiovascular mortality, fine particulate matter, ischemic heart disease, lung cancer, mortality, nitrogen dioxide

## Abstract

Background: Few European studies have investigated the effects of long-term exposure to both fine particulate matter (≤ 2.5 µm; PM_2.5_) and nitrogen dioxide (NO_2_) on mortality.

Objectives: We studied the association of exposure to NO_2_, PM_2.5_, and traffic indicators on cause-specific mortality to evaluate the form of the concentration–response relationship.

Methods: We analyzed a population-based cohort enrolled at the 2001 Italian census with 9 years of follow-up. We selected all 1,265,058 subjects ≥ 30 years of age who had been living in Rome for at least 5 years at baseline. Residential exposures included annual NO_2_ (from a land use regression model) and annual PM_2.5_ (from a Eulerian dispersion model), as well as distance to roads with > 10,000 vehicles/day and traffic intensity. We used Cox regression models to estimate associations with cause-specific mortality adjusted for individual (sex, age, place of birth, residential history, marital status, education, occupation) and area (socioeconomic status, clustering) characteristics.

Results: Long-term exposures to both NO_2_ and PM_2.5_ were associated with an increase in nonaccidental mortality [hazard ratio (HR) = 1.03 (95% CI: 1.02, 1.03) per 10-µg/m^3^ NO_2_; HR = 1.04 (95% CI: 1.03, 1.05) per 10-µg/m^3^ PM_2.5_]. The strongest association was found for ischemic heart diseases (IHD) [HR = 1.10 (95% CI: 1.06, 1.13) per 10-µg/m^3^ PM_2.5_], followed by cardiovascular diseases and lung cancer. The only association showing some deviation from linearity was that between NO_2_ and IHD. In a bi-pollutant model, the estimated effect of NO_2_ on mortality was independent of PM_2.5_.

Conclusions: This large study strongly supports an effect of long-term exposure to NO_2_ and PM_2.5_ on mortality, especially from cardiovascular causes. The results are relevant for the next European policy decisions regarding air quality.

Most of the available evidence linking long-term air pollution exposure with mortality comes from North American studies and is based on exposure contrasts between and within various communities (Abbey et al. 1999; Dockery et al. 1993; Gan et al. 2011; Lepeule et al. 2012; Lipsett et al. 2011; Pope et al. 2004; Puett et al. 2008). There are only a few European studies on the effects of long-term exposure to both fine particles (diameter ≤ 2.5 µm; PM_2.5_) and nitrogen dioxide (NO_2_) on all-cause and cause-specific mortality (Beelen et al. 2008; Filleul et al. 2005; Gehring et al. 2006; Hoek et al. 2002; Naess et al. 2007).

The estimated effects of long-term exposure to air pollution seem to be stronger for cardiovascular, respiratory, and lung-cancer mortality than for other causes of mortality (Beelen et al. 2008; Crouse et al. 2012; Dockery and Stone 2007; Gan et al. 2011; Lepeule et al. 2012; Lipsett et al. 2011; Pope et al. 2002, 2004), but the specific roles of PM_2.5_ and NO_2_, which both originate in urban areas (at least partially) from traffic and chemical transformation processes, have not been elucidated. Therefore, a recent review of the literature conducted by the Health Effects Institute (HEI) states that the evidence linking traffic air pollution and mortality is suggestive but not yet sufficient (HEI Panel on the Health Effects of Traffic-related Air Pollution 2010).

In 2013, the European Union (EU) will revise its main air pollution control policies (the EU air pollution directive 2008/50/EC). Hence, the European Commission has recently requested that the World Health Organization (WHO) respond to several scientific open questions [the REVIHAAP project, Evidence on Health Aspects of Air Pollution to Review EU Policies (WHO 2013)]. In particular, there is a need to better evaluate the form of the concentration–response functions of PM_2.5_, to assess the independent role of NO_2_ on PM_2.5_, and to establish the outcomes to be considered in health impact assessment studies.

The identification of population subgroups that may be particularly vulnerable to air pollution effects is an additional research concern. Some studies have suggested that sex, socioeconomic position, smoking, and health characteristics, which are usually treated as confounders, could modify exposure–mortality associations. For example, Chen et al. (2005) reported that coronary deaths were associated with increasing levels of PM_2.5_ in women but not men. Therefore, it has been suggested that the next generation of studies should identify the characteristics of subjects who are most susceptible to the effects of air pollution (Puett et al. 2008).

In the present study, we analyzed associations of NO_2_, PM_2.5_, and two GIS (geographic information system) indicators of traffic exposure (distance to heavy traffic roads with > 10,000 vehicles per day, and traffic intensity in a 150 m buffer) with cause-specific mortality in adults included in the Rome Longitudinal Study (RoLS; Cesaroni et al. 2010). We estimated the overall effect of each single pollutant and traffic indicator on mortality and examined the form of the concentration–response relationships. In addition, we investigated effect modification by personal characteristics (i.e., sex, age group, socioeconomic position) to identify potential susceptible subgroups.

## Methods

*The study cohort*. Rome is the largest Italian city, with a population of about 2.5 million inhabitants in a 1,290 km^2^ area at the 2001 Italian census (National Institute of Statistics 2001), with the majority of the population living within the large urban area, but also including suburban communities.

The RoLS is based on the 2001 census fixed cohort of Rome ascertained from the Municipal Register (Cesaroni et al. 2010). We included all residents ≥ 30 years of age on the census reference day (21 October 2001) who were not living in institutions (prisons, hospitals, or nursing homes) and who had resided in Rome for at least 5 years. Data were available on sex, age, place of birth and residential history and were obtained for additional variables (marital status, education, occupation) using record-linkage procedures under strict control to protect individual privacy.

We conducted a follow-up to determine vital status using the Rome Municipal Register during the period October 2001–December 2010. We retrieved information on deceased individuals and considered subjects as lost to follow-up when they moved out of the city. The underlying cause of death [coded according to the *International Classification of Diseases, 9th Revision* (ICD-9; WHO 1977)] for deceased subjects was retrieved from the Lazio regional health information system.

The RoLS is part of the National Statistical Program for the years 2011–2013 and was approved by the Italian Data Protection Authority.

*Air pollution exposure assessment*. We used a land use regression (LUR) model to estimate annual NO_2_ concentrations for each residence. The LUR model has been described previously (Cesaroni et al. 2012a). Briefly, in 2007 we measured NO_2_ concentrations using Ogawa passive samplers (Ogawa & Co. USA Inc., Pompano Beach, FL, USA) at 78 sites during three 1-week periods in February, May, and October. We assigned to each sampling location a single NO_2_ level, the mean of the three measurements. We used several land-use, GIS, and traffic variables to predict log NO_2_ levels in multivariable linear regression. The best-fitting regression model had a determination coefficient (*R*^2^) of 0.704. The model was validated using leave-one-out cross validation; the *R*^2^, adjusted *R*^2^, and root mean square error of the regression analysis between measured and estimated concentrations was 0.61, 0.61, and 5.38, respectively.

Residential exposure to PM_2.5_ was estimated using a 1 km–grid dispersion model [the flexible air quality regional model (FARM), a three-dimensional Eulerian model of the transport and multiphase chemistry of pollutants in the atmosphere (Gariazzo et al. 2007, 2011)]. [For further details on the dispersion model, its validation, and the comparison of the results from the NO_2_ and PM_2.5_ models with actual measurements are provided in Supplemental Material, pp. 2–3 (http://dx.doi.org/10.1289/ehp.1205862).]

We applied the estimated annual means from the 2007 NO_2_ LUR model and from the 2005 PM_2.5_ dispersion model to all addresses from October 1996 through December 2010. For each individual subject and each year of the follow-up, we calculated the average exposure since October 1996, weighted for the time of residence in each location.

We used two GIS indicators at the subjects’ residential address as proxy measures of exposure to traffic. The first was the distance to high traffic roads (HTRs; roads with > 10,000 vehicles per day, which we categorized as < 50, 50–100, 100–150, 150–250, and ≥ 250 m). The second was traffic intensity within the 150 m buffer zone around the home (the sum of the number of vehicles per day multiplied by the length of the roads in meters within the buffer) categorized in quintiles of the distribution. The size of the buffer was slightly larger than that used by Beelen et al. (2008). For the GIS variables, we used the address of the individual subjects at the baseline.

*Outcomes*. We analyzed mortality for nonaccidental causes (ICD-9 codes < 800), cardiovascular disease (ICD-9: 390–459), ischemic heart disease [IHD (ICD-9: 410–414)], cerebrovascular disease (ICD-9: 430–438), respiratory disease (ICD-9: 460–519), and lung cancer (ICD-9: 162).

*Covariates*. We considered age, sex, and several variables at the baseline as potential confounders: marital status (married, single, separated/divorced, or widowed), place of birth (Rome or other), level of education (university, high school, secondary, or primary), and occupation [top qualified non-manual employed (i.e., managers, university and high school professors, researchers); other non-manual employed; manual labor employed; other employed (i.e., armed forces and retail sales); housewife; unemployed; retired; other]. Some studies have shown that neighborhood socioeconomic level is associated with smoking, after accounting for individual education and occupation (Diez Roux et al. 2003). Therefore, we adjusted estimates for a five-level small-area (census block) socioeconomic position index that is based on 2001 census data in Rome (5,500 census blocks, average population of 500 subjects per block) and was derived based on a factor analysis including education, occupation, house ownership, family composition, crowding, and immigrant status (Cesaroni et al. 2010).

In addition, because data on lifestyles were unavailable, we adjusted a subset of models for preexisting comorbidities related to smoking habits or diet [diabetes (ICD-9 code 250), chronic obstructive pulmonary disease (COPD; ICD-9: 490–492, and 496), and hypertensive heart disease (ICD-9: 401–404)] that were identified based on the principal and up to five secondary diagnoses indicated on hospital discharges from October 1996 to October 2001 (Gan et al. 2011).

*Statistical analyses*. We investigated the correlation between exposure to NO_2_ and PM_2.5_ using Pearson’s correlation coefficient. We used Cox proportional hazards regression models [hazard ratios (HRs)] with time-dependent exposures and age as the time scale to estimate associations between air pollution exposure and cause-specific mortality. We first calculated HRs adjusted for sex only (model 1); then adjusted for individual covariates (marital status, place of birth, education, occupation) and the small area socioeconomic position indicator (model 2); and finally, we adjusted also for preexisting comorbidities [diabetes, hypertensive heart disease, and COPD (model 3)]. When analyzing respiratory mortality with model 3, we adjusted for diabetes and hypertensive heart disease only.

We estimated associations with the pollutants using several different scales: quintiles of the distributions, 10-µg/m^3^ increases, and interquartile range (IQR) increases. To estimate the overall effects of NO_2_ and PM_2.5_, we modeled each pollutant in turn (single-pollutant models), and estimated independent effects of each pollutant by including both PM_2.5_ and NO_2_ in the same multivariable Cox model (bi-pollutant model). We also adjusted single-pollutant models for traffic intensity and distance to an HTR.

In addition, we evaluated potential effect modification by including an interaction term between exposure (NO_2_ or PM_2.5_, in turn) and one effect modifier at a time [sex, age group, educational level, small area socioeconomic position, and residential stability (i.e., a binary variable indicating whether the subject had ever changed the residential address)] and used likelihood ratio tests to compare the fit of models with and without interaction terms.

We considered *p*-values < 0.05 as indication of statistical significance, and we performed Wald tests to test the trend across quintiles of exposures (treated as ordinal categorical variables coded using integer values 1–5).

Neighborhoods are usually inhabited by residents with similar characteristics (socioeconomic, health, access to services) and similar environmental exposures, which means that confounding and clustering in the association between exposure and mortality should be investigated (Crouse et al. 2012).

In a sensitivity analysis, we performed a frailty model to investigate the role of both neighborhood and district (Rome is divided into 94 neighborhoods and into 19 districts).

We explored the shape of relationships between exposures and outcomes by replacing the linear term in the base model with natural splines with 2, 3, or 4 degrees of freedom (df) (Eisen et al. 2004), which capture potential nonlinearity in the data without overfitting. We used the Bayesian information criterion (BIC) and the likelihood ratio test to compare the relative goodness of fit of the models.

We used STATA10 (StataCorp, College Station, TX, USA) for all statistical analyses with the exception of the frailty models and spline plots, for which we used R (R Foundation, Vienna, Austria). Because R was not able to deal with a large number of records for the amount of computer memory available, for the spline and frailty analyses we studied a 20% random sample of the study population and used fixed time-weighted exposures between October 1996 and October 2001. We applied the appropriate weights to natural spline models to plot effects for the entire population.

## Results

**Figure 1:**
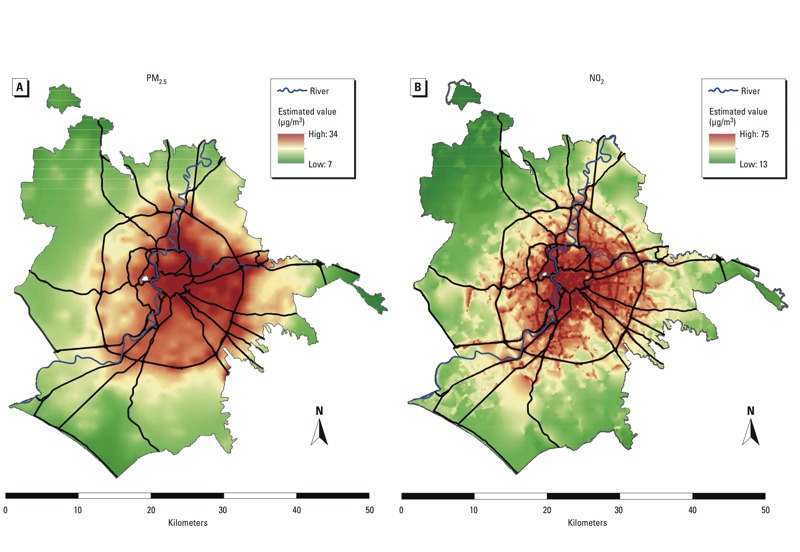
Maps of the concentrations of PM_2.5_ (*A*) and NO_2_ (*B*) in Rome. The NO_2_ map (adapted from Cesaroni et al. 2012a) was obtained using a natural neighbor interpolation method of all the estimated values at the study population’s residential addresses.

A total of 1,265,058 residents were included in the study. The average exposure levels of the population [mean (SD, range, 25th percentile, 50th percentile, 75th percentile)] at baseline were 43.6 µg/m^3^ (8.4, 13.0–75.2, 38.5, 44.5, 49.2) for NO_2_, and 23.0 µg/m^3^ (4.4, 7.2–32.1, 20.3, 23.9, 26.0) for PM_2.5_. The average distance to an HTR was 232 m (224, 2–946, 80, 165, 308), and the average traffic intensity within a 150 m buffer zone was 4.1 × 10^6^ vehicles/m (5.3 × 10^6^, 0–88.9 × 10^6^, 0.6 × 10^6^, 5.4 × 10^6^, 5.5 × 10^6^). We found a high correlation between NO_2_ and PM_2.5_ exposures (0.79). Figure 1 maps the concentrations of the two pollutants in Rome. The highest levels of air pollution are in the city center and in the eastern part of Rome. The resolution of the exposure model for NO_2_ is clearly higher than for PM_2.5_.

From October 2001 to December 2010 (average length of follow-up: 8.3 years), 9.5% of the study population emigrated, and 12% died. There were 144,441 nonaccidental deaths (95.8% of all deaths), and the crude mortality rate (CMR) was 13.8 per 10,000 person-years. Cardiovascular causes were responsible for 40% of all deaths (CMR = 5.8) [including IHD (15% of deaths, CMR = 2.2) and cerebrovascular diseases (9% of deaths, CMR = 1.3)]; respiratory diseases and lung cancer accounted for 6% (CMR = 0.8) and 8% of all deaths (CMR = 1.2), respectively. The majority of the cohort (75.3%) did not change address from October 1996 to the end of follow-up, whereas a change in address within the city was registered for the other 311,728 residents.

Residents with higher levels of NO_2_ exposure were older, better educated, and more likely to live alone, closer to HTRs, and more exposed to traffic compared with residents who had lower levels of exposure [see Supplemental Material, Table S1 (http://dx.doi.org/10.1289/ehp.1205862)].

*Air pollution and mortality*. Although there was little or no evidence of an association between any of the indices of exposure and nonaccidental, cardiovascular disease, or IHD mortality based on the crude model (model 1, adjusted for sex only), there was strong evidence of an association between all exposure indicators and these outcomes when we adjusted for personal characteristics and area-based socioeconomic position (model 2) (Table 1). The variables most responsible for the differences between the two models were education, occupation, and area-based socioeconomic position index (data not shown). The magnitude of the estimated effects on nonaccidental mortality was similar for NO_2_ and PM_2.5_, with a gradual increase in mortality across the quintiles of NO_2_ and PM_2.5_, as well as categories of traffic intensity and distance to HTRs. Although the *p*_trend_ was < 0.05 in the association between proximity to HTRs and mortality, only those living < 50 m from an HTR had a statistically significant higher mortality risk compared with those living ≥ 250 m from an HTR. Associations with the different exposure indexes followed similar patterns for cardiovascular disease and IHD mortality, and the estimated effects were stronger for IHD mortality than all other causes.

**Table 1:** Adjusted HRs (95% CI) of nonaccidental, cardiovascular, and IHD mortality according to different air pollution exposure indices, Rome 2001–2010.

Exposure	Nonaccidental causes (n = 144,441)			Cardiovascular disease (n = 60,318)			IHD (n = 22,562)		
	Cases	HRa (95% CI)	HRb (95% CI)	Cases	HRa (95% CI)	HRb (95% CI)	Cases	HRa (95% CI)	HRb (95% CI)
Quintiles of NO2c									
Q1	21,496	1.00	1.00	8,400	1.00	1.00	3,181	1.00	1.00
Q2	23,521	1.00 (0.98, 1.02)	1.04 (1.02, 1.06)	9,443	1.00 (0.97, 1.03)	1.04 (1.01, 1.08)	3,651	1.04 (0.99, 1.09)	1.09 (1.04, 1.14)
Q3	30,272	1.00 (0.98, 1.01)	1.05 (1.03, 1.07)	12,647	0.99 (0.96, 1.02)	1.06 (1.03, 1.09)	4,678	1.01 (0.96, 1.04)	1.08 (1.03, 1.13)
Q4	32,820	1.00 (0.98, 1.01)	1.06 (1.04, 1.07)	14,090	0.99 (0.96, 1.01)	1.06 (1.03, 1.09)	5,183	1.02 (0.98, 1.07)	1.10 (1.05, 1.15)
Q5	36,332	1.01 (0.99, 1.03)	1.07 (1.05, 1.09)	15,738	1.00 (0.98, 1.03)	1.07 (1.04, 1.10)	5,869	1.06 (1.01, 1.10)	1.13 (1.08, 1.19)
ptrend		0.184	< 0.001		0.994	< 0.001		0.041	< 0.001
10µg/m3 NO2		1.01 (1.00, 1.01)	1.03 (1.02, 1.03)		1.00 (0.99, 1.01)	1.03 (1.02, 1.04)		1.02 (1.00, 1.03)	1.05 (1.03, 1.06)
IQR NO2 (10.7 µg/m3)		1.01 (1.00, 1.01)	1.03 (1.02, 1.04)		1.00 (0.99, 1.01)	1.03 (1.02, 1.04)		1.02 (1.00, 1.04)	1.05 (1.03, 1.07)
Quintiles of PM2.5d									
Q1	22,432	1.00	1.00	8,878	1.00	1.00	3,339	1.00	1.00
Q2	25,657	0.98 (0.97, 1.00)	1.03 (1.01, 1.05)	10,238	0.95 (0.93, 0.98)	1.01 (0.98, 1.04)	3,925	0.99 (0.95, 1.04)	1.06 (1.01, 1.11)
Q3	28,109	0.98 (0.96, 1.00)	1.05 (1.03, 1.06)	11,560	0.95 (0.92, 0.98)	1.02 (1.00, 1.05)	4,346	0.99 (0.94, 1.03)	1.08 (1.03, 1.13)
Q4	32,194	1.01 (0.99, 1.03)	1.04 (1.03, 1.06)	13,823	1.01 (0.98, 1.04)	1.05 (1.02, 1.08)	5,085	1.04 (0.99, 1.08)	1.08 (1.03, 1.13)
Q5	36,049	1.00 (0.98, 1.01)	1.05 (1.03, 1.07)	15,819	1.00 (0.98, 1.03)	1.07 (1.04, 1.10)	5,867	1.05 (1.00, 1.09)	1.13 (1.08, 1.18)
ptrend		0.153	< 0.001		0.006	< 0.001		< 0.001	< 0.001
10 µg/m3 PM2.5		1.01 (1.00, 1.02)	1.04 (1.03, 1.05)		1.03 (1.01, 1.05)	1.06 (1.04, 1.08)		1.06 (1.02,1.09)	1.10 (1.06, 1.13)
IQR PM2.5 (5.8 µg/m3)		1.01 (1.00, 1.01)	1.02 (1.02, 1.03)		1.02 (1.00, 1.03)	1.04 (1.03, 1.05)		1.03 (1.01, 1.05)	1.06 (1.04, 1.07)
Distance to HTR (m)									
≥ 250	41,274	1.00	1.00	16,668	1.00	1.00	6,316	1.00	1.00
150–250	30,537	0.98 (0.97, 0.99)	0.99 (0.98, 1.01)	12,824	0.98 (0.95, 1.00)	0.99 (0.97, 1.02)	4,810	0.99 (0.95, 1.03)	1.01 (0.97, 1.05)
100–150	22,683	0.99 (0.97, 1.01)	1.01 (0.99, 1.02)	9,520	0.98 (0.96, 1.00)	1.00 (0.97, 1.02)	3,491	0.98 (0.94, 1.02)	0.99 (0.95, 1.04)
50–100	22,078	0.99 (0.97, 1.01)	1.01 (0.99, 1.03)	9,255	0.97 (0.94, 0.99)	0.99 (0.97, 1.02)	3,479	0.99 (0.95, 1.04)	1.02 (0.98, 1.06)
< 50	27,869	1.00 (0.99, 1.02)	1.02 (1.00, 1.01)	12,051	1.01 (0.98, 1.03)	1.03 (1.01, 1.05)	4,466	1.02 (0.98, 1.06)	1.05 (1.01, 1.09)
ptrend		0.688	0.004		0.953	0.043		0.385	0.034
Quintiles of traffic intensity within 150 me									
Q1	23,038	1.00	1.00	9,149	1.00	1.00	3,551	1.00	1.00
Q2	27,857	1.00 (0.98, 1.02)	1.02 (1.00, 1.04)	11,461	0.99 (0.96, 1.02)	1.02 (0.99, 1.04)	4,275	0.97 (0.93, 1.02)	1.00 (0.95, 1.04)
Q3	29,034	1.01 (0.99, 1.02)	1.03 (1.01, 1.05)	12,076	1.00 (0.97, 1.02)	1.03 (1.00, 1.05)	4,469	0.98 (0.94, 1.03)	1.01 (0.97, 1.04)
Q4	31,447	1.00 (0.98, 1.02)	1.03 (1.01, 1.05)	13,400	0.99 (0.97, 1.02)	1.03 (1.00, 1.06)	5,029	1.00 (0.96, 1.05)	1.04 (1.00, 1.09)
Q5	33,065	1.01 (0.99, 1.02)	1.04 (1.03, 1.06)	14,232	1.01 (0.98, 1.03)	1.05 (1.02, 1.07)	5,238	1.00 (0.96, 1.05)	1.04 (1.00, 1.09)
ptrend		0.218	< 0.001		0.570	0.001		0.421	0.009
aAdjusted for sex. bAdjusted for sex, marital status, place of birth, education, occupation, and area-based socioeconomic position. cQuintiles of NO2: Q1, ≤ 36.5; Q2, 36.5–42.7; Q3, 42.7–46.2; Q4, 46.2–50.4; Q5, > 50.4 µg/m3. dQuintiles of PM2.5: Q1, ≤ 19.4; Q2, 19.4–22.5; Q3, 22.5–24.8; Q4, 24.8–26.8; Q5, > 26.8 µg/m3. eQuintiles of traffic intensity (×106): Q1, < 0.25; Q2, 0.25–1.63; Q3, 1.63–3.23; Q4, 3.23–6.66; Q5, ≥ 6.66.									

Table 2 shows the results of cerebrovascular, respiratory, and lung-cancer mortality. There was evidence of an association between PM_2.5_ exposure and cerebrovascular mortality, with an 8% higher risk per 10-µg/m^3^ PM_2.5_ (model 2; 95% CI: 1.04, 1.13), but associations with NO_2_ and proxy measures of traffic exposure were weaker and not statistically significant. There was some evidence of an effect of NO_2_ and traffic intensity on respiratory disease mortality. There was strong evidence of an association between lung-cancer mortality and both NO_2_ and PM_2.5_, but not with proxy measures of traffic exposure.

**Table 2:** Adjusted HRs (95% CI) of mortality according to different air pollution exposure indices, Rome 2001–2010.

Exposure	Cerebrovascular disease (n = 13,576)			Respiratory disease (n = 8,825)			Lung cancer (n = 12,208)		
	Cases	HRa (95% CI)	HRb (95% CI)	Cases	HRa (95% CI)	HRb (95% CI)	Cases	HRa (95% CI)	HRb (95% CI)
Quintiles of NO2c									
Q1	1,935	1.00	1.00	1,242	1.00	1.00	2,008	1.00	1.00
Q2	2,141	0.97 (0.91, 1.03)	1.02 (0.96, 1.09)	1,412	1.01 (0.94, 1.09)	1.07 (0.99, 1.15)	2,187	1.04 (0.98, 1.11)	1.07 (1.01, 1.14)
Q3	2,830	0.94 (0.89, 1.00)	1.01 (0.96, 1.08)	1,798	0.95 (0.88, 1.02)	1.02 (0.95, 1.10)	2,568	1.05 (0.99, 1.11)	1.09 (1.03, 1.16)
Q4	3,151	0.94 (0.88, 0.99)	1.01 (0.96, 1.08)	2,043	0.97 (0.90, 1.04)	1.05 (0.97, 1.13)	2,610	1.05 (0.99, 1.11)	1.09 (1.03, 1.16)
Q5	3,519	0.95 (0.90, 1.00)	1.03 (0.97, 1.09)	2,330	1.01 (0.94, 1.08)	1.08 (1.00, 1.16)	2,835	1.07 (1.01, 1.13)	1.11 (1.05, 1.18)
ptrend		0.040	0.459		0.967	0.097		0.054	0.002
10 µg/m3 NO2		0.98 (0.96, 1.00)	1.01 (0.99, 1.03)		1.00 (0.98, 1.03)	1.03 (1.00, 1.06)		1.03 (1.00, 1.05)	1.04 (1.02, 1.07)
IQR NO2 (10.7 µg/m3)		0.98 (0.95, 1.00)	1.01 (0.99, 1.03)		1.01 (0.98, 1.03)	1.03 (1.00, 1.06)		1.03 (1.01, 1.06)	1.05 (1.02, 1.08)
Quintiles of PM2.5d									
Q1	2,018	1.00	1.00	1,319	1.00	1.00	2,090	1.00	1.00
Q2	2,228	0.91 (0.85, 0.96)	0.97 (0.91, 1.03)	1,542	0.96 (0.90, 1.04)	1.03 (0.96, 1.11)	2,268	1.01 (0.95, 1.07)	1.04 (0.98, 1.10)
Q3	2,577	0.92 (0.86, 0.97)	1.00 (0.94, 1.06)	1,744	0.96 (0.90, 1.03)	1.05 (0.98, 1.13)	2,397	1.03 (0.97, 1.10)	1.09 (1.02, 1.15)
Q4	3,114	0.98 (0.93, 1.04)	1.03 (0.97, 1.09)	1,953	0.96 (0.89, 1.03)	1.00 (0.93, 1.07)	2,611	1.05 (0.99, 1.11)	1.07 (1.01, 1.13)
Q5	3,639	0.99 (0.94, 1.05)	1.08 (1.02, 1.14)	2,267	0.97 (0.91, 1.04)	1.05 (0.97, 1.12)	2,842	1.03 (0.98, 1.10)	1.08 (1.02, 1.15)
ptrend		0.076	< 0.001		0.507	0.536		0.105	0.006
10 µg/m3 PM2.5		1.03 (0.99, 1.08)	1.08 (1.04, 1.13)		0.99 (0.94, 1.04)	1.03 (0.97, 1.08)		1.02 (0.98, 1.07)	1.05 (1.01, 1.10)
IQR PM2.5 (5.8 µg/m3)		1.02 (0.99, 1.04)	1.05 (1.02, 1.07)		0.99 (0.96, 1.02)	1.01 (0.99, 1.05)		1.01 (0.99, 1.04)	1.03 (1.01, 1.06)
Distance to HTR (m)									
≥ 250	3,721	1.00	1.00	2,458	1.00	1.00	3,782	1.00	1.00
150–250	2,908	0.98 (0.94, 1.03)	1.00 (0.96, 1.05)	1,848	0.95 (0.89, 1.01)	0.97 (0.91, 1.03)	2,552	0.98 (0.93, 1.03)	0.98 (0.93, 1.03)
100–150	2,131	0.97 (0.92, 1.02)	1.00 (0.94, 1.05)	1,356	0.95 (0.88, 1.01)	0.96 (0.90, 1.03)	1,894	1.00 (0.95, 1.06)	1.01 (0.96, 1.07)
50–100	2,111	0.98 (0.93, 1.03)	1.01 (0.95, 1.06)	1,413	1.00 (0.94, 1.07)	1.02 (0.96, 1.09)	1,790	0.99 (0.94, 1.05)	1.01 (0.95, 1.07)
< 50	2,705	1.00 (0.95, 1.05)	1.03 (0.98, 1.08)	1,750	0.99 (0.93, 1.05)	1.01 (0.95, 1.08)	2,190	0.98 (0.93, 1.03)	0.99 (0.94, 1.05)
ptrend		0.848	0.305		0.863	0.390		0.670	0.907
Quintiles of traffic intensity within 150 m									
Q1	2,067	1.00	1.00	1,320	1.00	1.00	2,179	1.00	1.00
Q2	2,632	1.00 (0.94, 1.05)	1.02 (0.97, 1.09)	1,673	1.00 (0.93, 1.07)	1.02 (0.95, 1.10)	2,437	1.01 (0.95, 1.07)	1.02 (0.96, 1.08)
Q3	2,686	0.97 (0.91, 1.02)	1.00 (0.95, 1.06)	1,796	1.03 (0.95, 1.10)	1.06 (0.99, 1.14)	2,433	1.00 (0.94, 1.06)	1.02 (0.96, 1.08)
Q4	3,015	0.97 (0.92, 1.03)	1.02 (0.96, 1.08)	1,917	0.98 (0.92, 1.05)	1.02 (0.95, 1.10)	2,567	1.01 (0.95, 1.07)	1.04 (0.98, 1.10)
Q5	3,176	0.97 (0.92, 1.03)	1.02 (0.97, 1.08)	2,119	1.04 (0.97, 1.11)	1.08 (1.00, 1.15)	2,592	1.00 (0.95, 1.06)	1.03 (0.97, 1.09)
ptrend		0.231	0.584		0.439	0.065		0.865	0.300
aAdjusted for sex. bAdjusted for sex, marital status, place of birth, education, occupation, and area-based socioeconomic position. cQuintiles of NO2: Q1, ≤ 36.5; Q2, 36.5–42.7; Q3, 42.7–46.2; Q4, 46.2–50.4; Q5, > 50.4 µg/m3. dQuintiles of PM2.5: Q1, ≤ 19.4; Q2, 19.4–22.5; Q3, 22.5–24.8; Q4, 24.8–26.8; Q5, > 26.8 µg/m3. eQuintiles of traffic intensity (×106): Q1, < 0.25; Q2, 0.25–1.63; Q3, 1.63–3.23; Q4, 3.23–6.66; Q5, ≥ 6.66.									

Estimated associations with NO_2_ and PM_2.5_ were similar or slightly stronger for all outcomes when we also adjusted for preexisting comorbidity [see Supplemental Material, Table S2 (http://dx.doi.org/10.1289/ehp.1205862)].

Associations with 10-µg/m^3^ increases in NO_2_ and PM_2.5_ (performed on a 20% random sample) were similar when estimated using the standard Cox model (model 2), the frailty model with districts, and frailty model with neighborhoods [see Supplemental Material, Table S3 (http://dx.doi.org/10.1289/ehp.1205862)]. The effect estimates in the 20% sample were very similar to those obtained for the entire data set, with the only exception of the PM_2.5_–lung cancer association, which was clearly underestimated.

**Figure 2:**
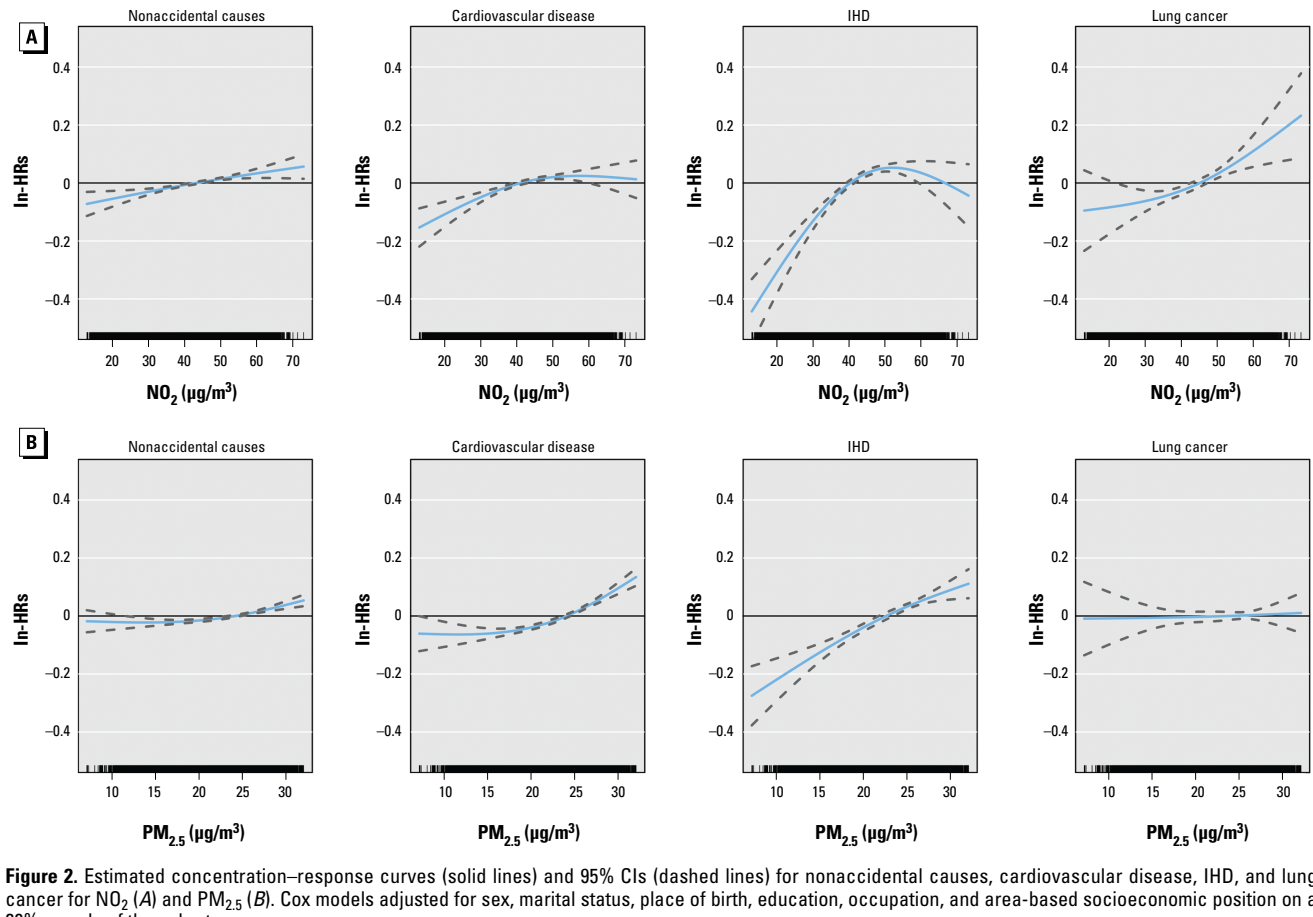
Estimated concentration–response curves (solid lines) and 95% CIs (dashed lines) for nonaccidental causes, cardiovascular disease, IHD, and lung cancer for NO_2_ (*A*) and PM_2.5_ (*B*). Cox models adjusted for sex, marital status, place of birth, education, occupation, and area-based socioeconomic position on a 20% sample of the cohort.

Figure 2 shows estimated concentration–response curves (natural splines, 2 df) for nonaccidental mortality, cardiovascular, IHD, and lung-cancer mortality for NO_2_ (Figure 2A) and PM_2.5_ (Figure 2B) based on a 20% random sample of the study population. In general, the results showed no evidence of deviation from linearity (based on BIC), with the only exception being the association between NO_2_ exposure and IHD mortality (likelihood ratio test comparing the linear and the spline model with 2 df gave a *p*-value = 0.028, although with very similar BIC). Results were similar for natural splines with 3 or 4 df (data not shown).

Despite the high correlation between the two pollutants, the estimated effect of a 10-µg/m^3^ increase in NO_2_ on nonaccidental mortality was still statistically significant when adjusted for PM_2.5_ in a bi-pollutant model [model 2; HR = 1.02 (95% CI: 1.01, 1.03)]. In contrast, the estimated effect of PM_2.5_ decreased when adjusted for NO_2_ [HR = 1.01 (95% CI: 0.99, 1.02) for a 10-µg/m^3^ increase in PM_2.5_ compared with HR = 1.04 (95% CI: 1.03, 1.05) based on the single pollutant model]. Adjusting for proximity to an HTR or traffic intensity in separate models of NO_2_ and PM_2.5_ did not change estimates for either pollutant (data not shown).

**Figure 3:**
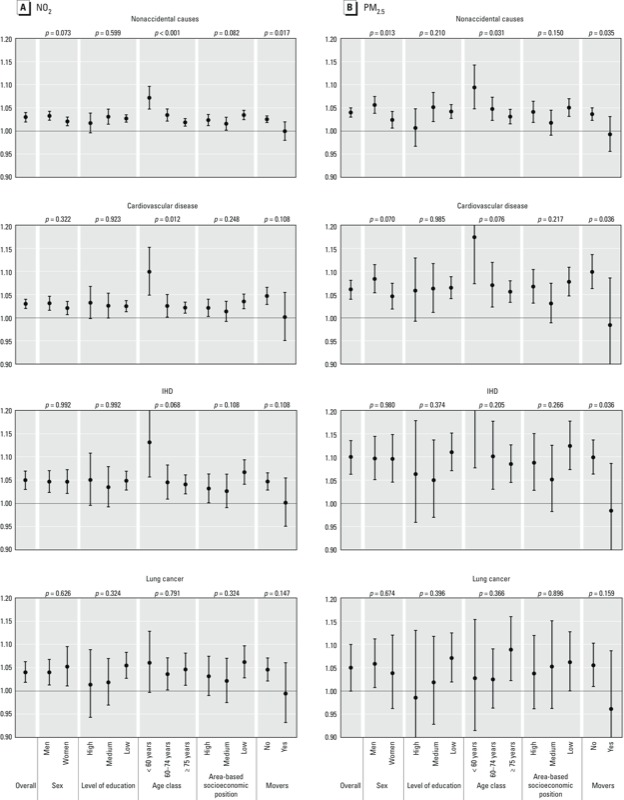
Adjusted HRs (95% CIs) and *p*-values for interaction for cause-specific mortality per 10‑µg/m^3^ elevation in NO_2_ (*A*) and PM_2.5_ concentrations (*B*), by population characteristics and cause of death.

Figure 3 presents the adjusted HRs, 95% CIs, and *p*-values for interaction (likelihood ratio test) for nonaccidental, cardiovascular, IHD, and lung-cancer mortality per 10-µg/m^3^ NO_2_ and PM_2.5_, by sex, level of education (high = university, middle = high school, low = secondary and primary school), age group, area-based socioeconomic position, and residential stability (movers: those who changed residence during the study). There was some suggestion of effect modification by age (with < 60-year-olds at higher risk than ≥ 75-year-olds), by residential stability (with non-movers at higher risk than movers), and by sex (with men at higher risk than women) for nonaccidental and cardiovascular mortality (for PM_2.5_ only).

## Discussion

We found statistically significant positive associations between long-term exposure to NO_2_ and PM_2.5_ and nonaccidental, cardiovascular, IHD, and lung-cancer mortality in the adult population of Rome. In addition, exposure to PM_2.5_ was associated with cerebrovascular mortality, whereas NO_2_ exposure was associated with respiratory mortality. Proximity to HTRs and high traffic intensity were associated with nonaccidental, cardiovascular, and IHD mortality. Despite the high correlation of the pollutants, NO_2_ was significantly associated with mortality when adjusted for PM_2.5_, although the estimated effect of PM_2.5_ was no longer significant. There was no evidence of deviation from linearity of the effects of either NO_2_ or PM_2.5_ on nonaccidental, cardiovascular, and lung-cancer mortality. The estimated effects on nonaccidental mortality tended to be stronger in males, younger subjects (< 60 years of age), and non-movers.

Average exposure of the cohort was slightly higher than in other study populations from Europe or North America, but near the values being discussed as potential European standards (40 µg/m^3^ for NO_2_ and 20 µg/m^3^ for PM_2.5_). The mean concentrations of NO_2_ and PM_2.5_ (in Rome: 44 µg/m^3^ and 23 µg/m^3^, respectively) ranged from 32.1 and 4.1 µg/m^3^ in Canada (Gan et al. 2011) to 39 µg/m^3^ NO_2_ in Germany (Gehring et al. 2006), and to 28.3 µg/m^3^ PM_2.5_, respectively, in the Netherlands (Beelen et al. 2008).

The associations we found for the selected causes of mortality were comparable with, but slightly lower than, those reported in other European and North American settings (Crouse et al. 2012). The 4% (95% CI: 3, 5%) higher risk of nonaccidental mortality per 10-µg/m^3^ PM_2.5_ in Rome was comparable to the 6% higher risk (95% CI: 3, 9%) reported based on a meta-analysis of five studies (Abbey et al. 1999; Beelen et al. 2008; Gehring et al. 2006; Laden et al. 2006; Pope et al. 2002), whereas the 3% (95% CI: 2, 3%) estimated increase in risk per 10-µg/m^3^ NO_2_ was lower than the meta-analytic estimate of 6% (95% CI: 4, 8%) based on four European studies (Beelen et al. 2008; Filleul et al. 2005; Gehring et al. 2006; Nafstad et al. 2004) [data reported in Cesaroni et al. (2012b)].

As expected, associations with IHD and cardiovascular mortality were stronger than with other causes of death (Crouse et al. 2012; Jerrett et al. 2009; Lepeule et al. 2012). Linear association between the pollutant exposures and cause-specific mortality were reported in some previous studies (Crouse et al. 2012; Gan et al. 2011). The shapes of the NO_2_ curves were similar to estimates reported for a study population in Olso, Norway (Naess et al. 2007).

Although stronger estimated effects for non-movers may simply reflect improved exposure estimation, evidence of higher risks in men compared with women deserves additional attention. Evidence of sex differences in susceptibility to air pollution is controversial. In the United States, an association between exposure to PM_10_ (particles with a diameter ≤ 10 µm)and mortality was reported for women in the Nurses’ Health Study (Puett et al. 2008) but not for men in the Health Professionals Follow-Up Study (Puett et al. 2011). However, Gan et al. (2011) reported strong evidence of an effect of black carbon on coronary heart disease mortality (after adjusting for NO_2_ and PM_2.5_) in men but not women. We estimated the strongest effects in the youngest age group of our population (< 60 years), consistent with a previous study (Naess et al. 2007).

Our study has several strengths. It is the largest European cohort study of the effects of both NO_2_ and PM_2.5_ and provides the statistical power to detect the effects of different indices of exposure on mortality. Residential history and several individual characteristics were available, and we had estimates of both NO_2_ and PM_2.5_ at the residences of all subjects.

This study has some limitations. The RoLS is a cohort built on administrative data and information on individual risk factors such as smoking habits, diet, alcohol consumption, and obesity were not available. As previously done in the literature, we adjusted the models for preexisting diabetes, COPD, and hypertensive heart disease—conditions which share the lifestyle risk factors cited (Gan et al. 2011). We adjusted also for small-area socioeconomic position, which could be a predictor of smoking habits independent of personal characteristics (Diex Roux et al. 2003). The adjustment for preexisting conditions might have led to an underestimation of the effect, because the comorbidities might act as intermediate variables (Gan et al. 2011). To further investigate the role of smoking, we selected 7,845 adult subjects from the study population for whom information on smoking habits was available from another investigation (the Italian Studies on Respiratory Disorders in Childhood and Environment (SIDRIA) study; Cesaroni et al. 2008). Once we adjusted for all covariates used in model 2 in a logistic regression model predicting ever smoking, there was no evidence of an association between exposure to NO_2_ or PM_2.5_ and ever smoking (all odds ratios close to 1.0), indicating that smoking is unrelated to the exposures and thus an unlikely confounder. Moreover, when we added smoking status in a survival analysis (model 2) restricted only to SIDRIA participants, the association between the air pollution exposures and nonaccidental mortality did not change (data not shown).

To analyze frailty and concentration–response curves we had to use a 20% random sample of the population, but these alternate models provided only slightly different estimates of the effects for nonaccidental, cardiovascular, and IHD mortality. Therefore, we expect that frailty analyses of the entire population would be comparable. On the other hand, PM_2.5_ effect estimates for lung cancer based on the 20% sample were quite different (close to unity) from estimates based on the entire population, and therefore the relative spline plot should be interpreted cautiously.

To estimate NO_2_ and PM_2.5_ exposure we used both an LUR model based on measurements carried out on 2007 and a dispersion model based on simulation for the year 2005, respectively. Both models were independently validated [see Supplemental Material, pp. 2–3 (http://dx.doi.org/10.1289/ehp.1205862)]. PM_2.5_ and NO_2_ were highly correlated, but PM_2.5_ estimates had a lower resolution than estimates for NO_2_. We are fairly confident that the spatial gradient of pollutants within the city remained stable over time. Rome is a city that changes very slowly, as two NO_2_ LUR models developed using measures taken 12 years apart showed very similar results both in terms of estimates of exposure of the population (the correlation was 0.96) and in their associations with natural mortality (Cesaroni et al. 2012a). We took into account the changes of address (and exposure) during the follow-up in the main time-dependent analyses. Conversely, we used time-weighted exposure for the 5 years before enrollment, without taking account of changes of address, for frailty models and spline curves.We have evaluated that the bias introduced in this way is negligible because the results on the entire population based on 1996–2001 average exposure were similar to those obtained with time-dependent exposure (data not shown).

## Conclusions

Long-term exposure to NO_2_ and PM_2.5_ was associated with increased mortality in this large population-based cohort. We found the strongest associations with IHD, followed by cardiovascular and lung-cancer mortality. The estimated effect of NO_2_ persisted after adjusting for PM_2.5_, and the shapes of the concentration response for both pollutants showed no evidence of deviation from linearity for all causes except IHD. European policy decisions regarding environment and public health should be made with consideration of the specific scientific research results on the health effects of air pollution, such as those provided here.

## Supplemental Material

(471 KB) PDF
