# Supplementary material for: Long-Term Exposure to Urban Air Pollution and Mortality in a Cohort of More than a Million Adults in Rome
Source: Environ Health Perspect. 2013 Jan 8;121(3):324–31. doi: 10.1289/ehp.1205862 (PMC3621202; doi:10.1289/ehp.1205862)
Supplement: (471 KB) PDF [file ehp.1205862.s001.pdf]

Supplemental Material

Long-term Exposure to Urban Air Pollution and Mortality in a Cohort of More than A Million Adults in Rome

Giulia Cesaroni[1], Chiara Badaloni[1], Claudio Gariazzo[2], Massimo Stafoggia[1], Roberto Sozzi[3], Marina Davoli[1], and Francesco Forastiere[1]

[1]Department of Epidemiology, Lazio Regional Health Service, Rome, Italy
[2]Italian Workers' Compensation Authority (INAIL), Rome, Italy
[3]Regional Environmental Protection Agency, Rome, Italy

Table of Contents

$PM_{2.5}$ dispersion model **p. 2-3**

Table S1. Characteristics of Participants, Length of Follow-up, and Exposure Indicators at baseline. Rome 2001-2010. **p. 4**

Table S2. Adjusted Hazard Ratios (HRs, 95%CI) of Mortality According to Different Air Pollution Exposure Indices and Adjusting for Comorbidity at Baseline. Rome 2001-2010. **p. 5**

Table S3. Adjusted Hazard Ratios (HRs, 95%CI) for Cause-Specific Mortality per 10μg/m^3 NO_2 and 10μg/m^3 $PM_{2.5}$ using Standard Cox Model and Frailty Models on a 20% Random Sample of the Study Population (253,012 subjects). Rome 2001-2010 **p. 6**

References **p. 7-8**

$PM_{2.5}$ dispersion model

Exposure to $PM_{2.5}$ at residence was predicted by means of a chemical transport model (the flexible air quality regional model - FARM) (Silibello et al. 2008a, 2008b). FARM is a three-dimensional Eulerian model dealing with the transport, chemical transformation and deposition of multiphase pollutants in the atmosphere. Simulations of emission, dispersion, transformation and deposition of pollutants were conducted over the whole year 2005 in the regional domain (Lazio), covering a significant portion of Central Italy (66x58 cells, 4km x 4km). The target domain included the Rome urban area (61x61 cells, 1km x 1km) using a nested approach. The modeling system was built to provide hourly concentrations of pollutants defined in the current Italian legislation. Diffuse emissions were estimated using the National Italian inventory (APAT 2000) projected to the year of interest using national trends differentiated for each pollutant and activity. As for the traffic emissions in the city of Rome, a traffic model has been used starting from socio-economic data, characteristics of mobility networks (road and public transport) of the study area and traffic flow data over the road network made up of 6000 links. The initial conditions of the domain were derived from climatological fields (monthly averaged daily values of gas and aerosol compounds) calculated from simulations carried out at national level within the MINNI project (Integrated National Model in support to the International Negotiation on air pollution, www.minni.org; Zanini et al. 2004). The model was first developed and validated for PM_{10} (Gariazzo et al. 2007; Gariazzo et al. 2011; Silibello et al. 2008b). Silibello and colleagues reported good agreement between observed and predicted PM_{10} annual mean, with an underestimation of observed levels at high traffic sites (Silibello et a. 2008b). The PM_{10} results (Gariazzo et al. 2011) indicated that the modeling system was able to reproduce the seasonal variability, with underestimation during the spring and summer seasons, possibly due to coarse dust Saharan episodes, and a good reproduction of observed data in winter and autumn seasons. When daily measures of $PM_{2.5}$ concentrations measured at an urban background monitoring station in Rome (Villa Ada) were compared with

modeled concentrations in the period January-July 2005, a good correlation was observed (correlation coefficient=0.83). Good agreement was also found between the observed and estimated $PM_{2.5}$ components (not shown here).

External validation of $PM_{2.5}$ and NO_2 models

To validate the spatial results of the LUR and dispersion models we used independent NO_2 and $PM_{2.5}$ measurements taken in the city during the 2010 survey (Cyrys et al. 2012; Eeftens et al. 2012). We measured $PM_{2.5}$ in 20 locations and NO_2 in 40 sites during three 2-weekly periods across the year to capture seasonal variations, and we calculated adjusted annual average exposure for each site.

We used 18 sites of the $PM_{2.5}$ measurements (we excluded the two regional background sites located outside Rome) and we compared the results with the values estimated with the dispersion model. The mean of the measured $PM_{2.5}$ concentrations was 19.9 µg/m^3 (sd 3.4) while the mean of the estimated concentrations from dispersion modeling was 23.2 µg/m^3 (sd 3.5). The correlation coefficient between estimated and measured $PM_{2.5}$ values was 0.64.

Similarly, we used 28 sites of NO_2 measurements (we excluded all the sites which were already used for the LUR model development and the two regional background sites located outside Rome), and we compared the values estimated from our LUR model. The mean of 2010 measured NO_2 concentrations was 44.8 µg/m^3 (sd 14.4) while the mean of the estimated concentrations from the LUR model was 44.0 µg/m^3 (sd 8.2). The correlation coefficient between estimated and measured NO_2 concentrations was 0.71.

Supplemental Material, Table S1. Characteristics of Participants, Length of Follow-up, and Traffic Exposure Indicators at baseline according to quintiles of NO_2 exposure. Rome 2001-2010

Characteristics of Participants (%)	Average length of follow-up (years)	Quintiles of NO_2 (range, µg/m^3)					Total
		<37	37-43	43-46	46-50	>50	
**N**	8.3	253,025	253,054	252,976	253,006	252,997	1,265,058
**Vital status**							
Alive at 31st Dec 2010	9.2	81.4	79.8	78.0	77.2	76.2	78.5
Emigrated	5.2	9.6	9.9	9.5	9.2	9.5	9.5
Deceased	4.7	9.1	10.3	12.5	13.6	14.3	12.0
**Sex**							
Male	8.2	47.4	46.4	45.4	44.5	44.0	45.5
Female	8.3	52.6	53.6	54.6	55.5	56.0	54.5
**Age at inclusion, years**							
< 60	8.7	68.7	65.0	59.5	57.2	56.4	61.3
60-75	8.2	23.9	25.8	27.8	28.2	28.2	26.8
≥ 75	6.3	7.4	9.2	12.7	14.6	15.4	11.9
**Marital status**							
Married	8.4	73.2	69.5	65.8	62.8	60.3	66.3
Single	8.4	11.7	13.6	15.3	17.0	18.7	15.3
Separated/Divorced	8.4	6.6	7.1	6.8	7.1	7.5	7.0
Widowed	7.3	8.5	9.9	12.1	13.1	13.5	11.4
**Place of birth**							
Rome	8.5	49.9	51.4	51.7	52.6	53.3	51.8
Other	8.0	50.1	48.6	48.3	47.4	46.7	48.2
**Level of education**							
University	8.5	10.3	15.1	17.3	19.1	19.4	16.2
High school	8.5	31.7	33.2	33.2	33.1	33.5	32.9
Secondary school	8.3	30.2	26.8	25.1	24.0	23.7	25.9
Primary school or less	7.8	27.9	24.9	24.5	23.9	23.4	24.9
**Occupational status**							
Employed NM I	8.7	10.5	13.2	13.8	14.8	15.1	13.5
Employed NM II	8.7	15.2	16.8	16.1	15.8	15.8	15.9
Employed M	8.6	13.7	10.7	9.1	8.3	7.8	9.9
Employed, other	8.6	7.8	7.0	6.2	5.7	5.8	6.5
Housewives	8.3	22.5	20.8	21.2	20.7	19.9	21.0
Unemployed	8.6	6.0	5.6	4.6	4.3	4.2	5.0
Retired	7.6	19.4	21.4	24.4	25.7	26.3	23.4
Other condition	7.5	4.9	4.6	4.5	4.8	5.1	4.8
**Area-based socioeconomic position**							
Very High	8.3	7.4	20.2	23.1	27.3	21.0	19.8
High	8.2	12.6	17.9	21.2	22.6	27.5	20.4
Medium	8.3	17.8	17.9	19.0	20.0	25.6	20.1
Low	8.3	30.9	19.3	19.0	15.6	17.1	20.4
Very Low	8.3	31.2	24.8	17.7	14.4	8.8	19.4
**Comorbidity conditions**							
Diabetes	6.5	2.2	2.3	2.3	2.3	2.3	2.3
COPD	6.1	1.9	2.1	2.1	2.0	2.0	2.0
Hypertensive heart disease	7.0	6.1	6.2	6.3	6.5	6.6	6.3
**Change of residence within the city**							
No	8.1	74.8	74.3	75.9	75.6	76.2	75.4
Yes	8.7	25.2	25.7	24.1	24.4	23.8	24.6
**Exposure baseline indicators (mean, sd)**							
Distance to High Traffic Roads (m)		457±300	264±186	222±149	146±111	69±76	232±224
Traffic intensity within 150 m (vehicles*m/10^6)		1.3 ± 2.3	2.1 ± 3.6	2.8 ± 4.3	4.6 ± 4.7	9.5 ± 6.5	4.1 ± 5.4
$PM_{2.5}$ (µg/m^3)[a]		17 ± 3	22 ± 3	25 ± 3	26 ± 3	26 ± 2	23 ± 5

[a]$PM_{2.5}$ quintiles at baseline were: ≤19.4,19.4-22.5, 22.5-24.8, 24.8-26.8,>26.8 µg/m^3

Supplemental Material, Table S2. Adjusted Hazard Ratios (HRs, 95%CI) of Mortality According to Different Air Pollution Exposure Indices and Adjusting for Comorbidity at Baseline. Rome 2001-2010

**Exposure**	**Non-accidental Causes (N=144,441)**	**Cardiovascular Disease (N=60,318)**	**Ischemic Heart Disease (N=22,562)**	**Cerebrovascular Disease (N=13,576)**	**Respiratory Disease (N=8,825)**	**Lung Cancer (N=12,208)**
	**HR(95% CI)**	**HR(95% CI)**	**HR(95% CI)**	**HR(95% CI)**	**HR(95% CI)**	**HR(95% CI)**
**Quintiles of NO_2**						
Q1	1.00	1.00	1.00	1.00	1.00	1.00
Q2	1.03 (1.01, 1.05)	1.03 (1.00, 1.07)	1.08 (1.02, 1.13)	1.01 (0.95, 1.08)	1.06 (0.98, 1.14)	1.07 (1.00, 1.14)
Q3	1.06 (1.04, 1.07)	1.06 (1.03, 1.09)	1.09 (1.04, 1.14)	1.02 (0.96, 1.08)	1.02 (0.95, 1.10)	1.09 (1.03, 1.16)
Q4	1.06 (1.04, 1.08)	1.07 (1.04, 1.10)	1.11 (1.07, 1.17)	1.02 (0.96, 1.08)	1.05 (0.98, 1.13)	1.10 (1.03, 1.16)
Q5	1.08 (1.06, 1.10)	1.08 (1.05, 1.11)	1.15 (1.10, 1.20)	1.04 (0.98, 1.10)	1.08 (1.01, 1.16)	1.12 (1.05, 1.19)
p-trend	*<0.001*	*<0.001*	*<0.001*	*0.223*	*0.056*	*<0.001*
10µg/m^3 NO_2	1.03 (1.03, 1.04)	1.03 (1.02, 1.04)	1.05 (1.04, 1.07)	1.01 (0.99, 1.03)	1.03 (1.01, 1.06)	1.05 (1.02, 1.07)
IQR NO_2(10.7µg/m^3)	1.04 (1.03, 1.04)	1.04 (1.02, 1.05)	*1.06 (1.04, 1.08)*	1.01 (0.99, 1.04)	1.04 (1.01, 1.07)	1.05 (1.03, 1.08)
**Quintiles of $PM_{2.5}$**						
Q1	1.00	1.00	1.00	1.00	1.00	1.00
Q2	1.02 (1.00, 1.04)	1.00 (0.97, 1.03)	1.05 (1.00, 1.10)	0.96 (0.90, 1.02)	1.03 (0.95, 1.11)	1.03 (0.97, 1.10)
Q3	1.05 (1.04, 1.07)	1.04 (1.01, 1.07)	1.09 (1.04, 1.14)	1.01 (0.95, 1.07)	1.06 (0.98, 1.12)	1.09 (1.03, 1.16)
Q4	1.05 (1.04, 1.07)	1.06 (1.03, 1.09)	1.10 (1.05, 1.15)	1.04 (0.98, 1.10)	1.01 (0.94, 1.08)	1.08 (1.02, 1.14)
Q5	1.07 (1.05, 1.09)	1.09 (1.06, 1.12)	1.15 (1.10, 1.20)	1.09 (1.03, 1.16)	1.06 (0.98, 1.13)	1.09 (1.03, 1.16)
p-trend	*<0.001*	*<0.001*	*<0.001*	*<0.001*	*0.310*	*0.001*
10µg/m^3 $PM_{2.5}$	*1.05 (1.04, 1.07)*	*1.08 (1.06, 1.10)*	*1.12 (1.08, 1.15)*	1.09 (1.05, 1.14)	1.03 (0.98, 1.09)	1.06 (1.02, 1.11)
IQR $PM_{2.5}$ (5.8µg/m^3)	*1.03 (1.02, 1.04)*	*1.05 (1.03, 1.06)*	*1.07 (1.05, 1.09)*	1.05 (1.03, 1.08)	1.02 (0.99, 1.05)	1.04 (1.01, 1.06)

HR Hazard Ratios adjusted for sex, marital status, place of birth, education, occupation, area-based socioeconomic position, and pre-existing conditions (diabetes, COPD, and hypertensive heart disease for all causes with the exception of respiratory diseases for which we considered diabetes and hypertensive heart disease)

Quintiles of NO_2: Q1 ≤36.5, Q2 36.5-42.7, Q3 42.7-46.2, Q4 46.2-50.4, Q5 >50.4 µg/m^3

Quintiles of $PM_{2.5}$: Q1 ≤19.4, Q2 19.4-22.5, Q3 22.5-24.8, Q4 24.8-26.8, Q5 >26.8 µg/m^3

Supplemental Material, Table S3. Adjusted Hazard Ratios (HRs, 95%CI) for Cause-Specific Mortality per 10µg/m^3 NO_2 and 10µg/m^3 $PM_{2.5}$ using Standard Cox Model and Frailty Models on a Random Sample of 20% of the Study population (253,012 subjects). Rome 2001-2010

Cause of Death	Standard Cox model (10 µg/m^3 NO_2)	Random effect - neighborhood (10 µg/m^3 NO_2)	Random effect - district (10 µg/m^3 NO_2)	Standard Cox model (10 µg/m^3 $PM_{2.5}$)	Random effect - neighborhood (10 µg/m^3 $PM_{2.5}$)	Random effect - district (10 µg/m^3 $PM_{2.5}$)
Non-Accidental Cause (N=28,905)						
HR (95%CI)	1.02 (1.01, 1.04)	1.02 (1.01, 1.04)	1.02 (1.01, 1.04)	1.04 (1.01, 1.07)	1.04 (1.01, 1.07)	1.04 (1.01, 1.07)
p-value (frailty)		0.280	0.230		0.280	0.270
Cardiovascular Disease (N=12,154)						
HR (95%CI)	1.03 (1.00, 1.05)	1.03 (1.00, 1.06)	1.03 (1.00, 1.05)	1.10 (1.06, 1.15)	1.11 (1.06, 1.16)	1.10 (1.06, 1.15)
p-value (frailty)		0.074	0.110		0.170	0.220
Ischemic Heart Disease (N=4,472)						
HR (95%CI)	1.07 (1.03, 1.11)	1.07 (1.02, 1.11)	1.06 (1.02, 1.11)	1.16 (1.08, 1.24)	1.16 (1.07, 1.25)	1.16 (1.08, 1.24)
p-value (frailty)		0.170	0.180		0.220	0.320
Cerebrovascular Disease (N=2,753)						
HR (95%CI)	1.01 (0.96, 1.06)	1.01 (0.95, 1.07)	1.01 (0.96, 1.07)	1.09 (0.99, 1.19)	1.08 (0.98, 1.19)	1.09 (0.98, 1.21)
p-value (frailty)		0.080	0.049		0.170	0.073
Respiratory Disease (N=1,761)						
HR (95%CI)	1.04 (0.98, 1.10)	1.04 (0.98, 1.10)	1.04 (0.98, 1.10)	1.04 (0.93, 1.16)	1.04 (0.93, 1.16)	1.04 (0.93, 1.16)
p-value (frailty)		0.840	0.850		0.630	0.850
Lung Cancer (N=2,407)						
HR (95%CI)	1.06 (1.00, 1.11)	1.06 (1.00, 1.11)	1.06 (1.00, 1.11)	1.01 (0.92, 1.11)	1.01 (0.92, 1.11)	1.01 (0.92, 1.11)
p-value (frailty)		0.830	0.830		0.830	0.830

HR Hazard Ratios adjusted for sex, marital status, place of birth, education, occupation, area-based socioeconomic position

References

APAT.2000.Agency for Environmental Protection and Technical Services. http://www.sinanet.apat.it/it/sinanet/bdemi/

CyrysJ, Eeftens M, Heinrich J, AmpeC, ArmengaudA,BeelenR, et al.2012.Variation of NO2 and NOx concentrations between and within 36 European studyareas: Results from the ESCAPE study. Atmospheric Environment, 62:374-390.

Eeftens M, Tsai M, Ampe C, Anwander B, Beelen R, Bellander T, et al. 2012. Spatial variation of PM2.5, PM10, PM2.5 absorbance and PMcoarse concentrations between and within 20 European study areas and the relationship with NO2 – Results of the ESCAPE project. Atmospheric Environment, 62:303-317.

Gariazzo C, Silibello C, Finardi S, Radice P, Piersanti A, Calori G, et al. 2007.A gas/aerosol air pollutants study over the urban area of Rome using a comprehensive chemical transport model. Atmospheric Environment, 41:7286-7303.

Gariazzo C, Hänninen O, Amicarelli A, Pelliccioni A, Silibello C, Sozzi R, et al. 2011. Integrated model for the estimation of annual, seasonal and episode PM10 exposures of children in Rome, Italy. Air Qual Atmos Health. 4:169-178.

Pession G, Magri T, Zublena M, Agnesod G, Genon G, Blanc L et al. 2008 Dust generation and dispersion (PM10 and PM2.5) in the Aosta Valley. Presentation at the 12th International Conference on Harmonisation within Atmospheric Dispersion Modelling for Regulatory Purposes Conference. Croatia. Available at: http://www.arpa.vda.it/allegati/Poster-Harmo12_4585.pdf (Accessed on the 7th November 2012).

Silibello C, Calori G, Brusasca G, Giudici A, Angelino E, Fossati G, et al. 2008. Modelling ofPM10 concentrations over Milano urban area using two aerosol modules.Environ Modell Softw. 23:333–43.

Silibello C, Brusasca G, Piersanti A, Radice P, Bolignano A, Sozzi R, Nussio R, Tasco C, Gariazzo C. 2008. Future emissions scenario analysis over Rome urban area using coupled traffic assignment and chemical transport models. Available at: http://www.harmo.org/conferences/Proceedings/_Cavtat/publishedSections/O_S2-02.pdf (Accessed on the 7th November 2012)

Zanini G, Monforti-Ferrario F, Ornelli P, Pignatelli T, Vialetto G, Brusasca G, et a. 2004. The MINNI Project. Proceedings of the 9th International Conference on Harmonisation within Atmospheric Dispersion Modelling for Regulatory Purposes (http://www.minni.org/pubblicazioni/HarmoIX_Garmisch%20-%20zanini%20et%20alii.pdf Accessed on the 7th November 2012)
